# The *Neisseria meningitidis* ADP-Ribosyltransferase NarE Enters Human Epithelial Cells and Disrupts Epithelial Monolayer Integrity

**DOI:** 10.1371/journal.pone.0127614

**Published:** 2015-05-21

**Authors:** Maria Valeri, Vanessa Zurli, Inmaculada Ayala, Antonino Colanzi, Lucia Lapazio, Daniela Corda, Marco Soriani, Mariagrazia Pizza, Silvia Rossi Paccani

**Affiliations:** 1 Vaccines & Diagnostics s.r.l.—a GSK company- Via Fiorentina 1, Siena, Italy; 2 Institute of Protein Biochemistry, National Research Council, Via P. Castellino 111, Naples, Italy; Universidad de Costa Rica, COSTA RICA

## Abstract

Many pathogenic bacteria utilize ADP-ribosylating toxins to modify and impair essential functions of eukaryotic cells. It has been previously reported that *Neisseria meningitidis* possesses an ADP-ribosyltransferase enzyme, NarE, retaining the capacity to hydrolyse NAD and to transfer ADP-ribose moiety to arginine residues in target acceptor proteins. Here we show that upon internalization into human epithelial cells, NarE gains access to the cytoplasm and, through its ADP-ribosylating activity, targets host cell proteins. Notably, we observed that these events trigger the disruption of the epithelial monolayer integrity and the activation of the apoptotic pathway. Overall, our findings provide, for the first time, evidence for a biological activity of NarE on host cells, suggesting its possible involvement in Neisseria pathogenesis.

## Introduction

*Neisseria meningitidis* is a Gram-negative, aerobic, non-motile, non-sporulating, usually encapsulated and piliated bacterium. It is restricted to humans and generally colonizes the nasopharynx of 8–20% of healthy individuals, however in a small proportion of infected patients, the bacterium crosses the mucosal barrier and reaches the bloodstream, giving rise to meningitis or fulminant septicaemia [1]. Masignani *et al*. have identified, through a profile-based computational approach, an ADP-ribosyltransferase protein, NarE, which shares structural homologies with *E*. *coli* heat-labile enterotoxin (LT) and cholera toxin (CT) [2]. NarE possesses both ADP-ribosylating and NAD-glycohydrolase activities, confirmed by the evidence that, in the presence of an ADP-ribose acceptor, NarE acts as a transferase whereas in the absence of the acceptor it acts as a NAD glycohydrolase [3]. Furthermore NarE undergoes auto-ADP-ribosylation [4].

Mono ADP-ribosylation is a post-translational modification of proteins, shared by eukaryotes and prokaryotes, which modulates protein function [5]. Mono-ADP-ribosyltransferases (ADPRTs) catalyze the transfer of a single ADP-ribose group of β-nicotinamide adenine dinucleotide (NAD^+^) to protein/peptide target acceptors with the release of nicotinamide (Nam) at the same time [6]. In pathogenic bacteria, proteins known to belong to this class of enzymes are generally classified as toxins since they alter or impair essential functions of host eukaryotic cells [7, 8]. On the basis of the ADPRTs targets, at least three groups of ADP-ribosylating toxins can be identified. One group causes ADP-ribosylation of G proteins. Members of this group are cholera toxin (CT) [9], *E*. *coli* heat-labile enterotoxin (LT) [10] and pertussis toxin (PT) [11], which, through modification of regulatory G proteins, impair signal transduction. The second group includes diphtheria toxin (DT) [12] and *Pseudomonas* exotoxin A (ExoA) [13] that target elongation factor 2 (EF-2), thus inhibiting protein synthesis. A large third group of bacterial toxins modulates actin cytoskeleton directly, by covalent modification of actin, as C2 toxin of *C*. *botulinum* [14], Iota toxin of *Clostridium* [15], VIP2 toxin of *Bacillus cereus* [16], and SpvB of *Salmonella* [17], or indirectly, by covalent modification of Rho GTPases, as C3 exoenzymes of *Clostridium*, *Bacillus cereus* and *S*.*aureus* [18, 19] exoenzyme S (ExoS) of *P*.*aeruginosa* [20].

Each group of toxins provides the bacterial pathogen with a selective advantage in modulating cell host response and resistance to infection, therefore they have been extensively characterized.

The *narE* gene is present only in a subset of hypervirulent clusters, ET-5 and Lineage 3 complexes, suggesting its involvement in *Neisseria meningitidis* pathogenesis [3]. However, no evidence of NarE toxic activity has been provided so far and its function remains to be fully elucidated. In the present report, we show that NarE targets Chang human epithelial cells. We demonstrated that NarE is internalized and gains access to the cytoplasm. Furthermore, through its ADP-ribosylating activity, NarE targets host cell proteins, alters epithelial monolayer integrity and initiates the apoptotic pathway responsible for cell death. Collectively, our data provide for the first time evidence of the biological role of this enzyme and suggest its potential contribution during colonization of upper respiratory tract and spreading of infection.

## Materials and Methods

### Cells, antibodies, reagents and recombinant proteins

Chang human epithelial cell line (HeLa contaminant) was purchased from the American Type Culture Collection (ATCC, CCL-20.2). Chang cells were maintained in minimum essential medium Eagle (MEM, Invitrogen Ltd, Paisley, UK) supplemented with 10% heat-inactivated fetal bovine serum (Invitrogen Ltd, Paisley, UK), 15mM L-glutamine and antibiotics. Cells were grown at 37°C in a humidified 5% CO_2_ atmosphere. In order to produce NarE polyclonal antiserum, CD1 mice were immunized with 10 μg of purified protein formulated with Al (OH) _3_, as an adjuvant. The recombinant protein was given intraperitoneally (day1), a second (day 21) and a third (day 35) booster doses were administered. Blood samples were taken on day 49. Antibody against cleaved caspase-3, anti-GAPDH and anti-Lamin were from Cell Signaling Technology (Beverly, MA). Antibody anti-ADAM10 was from Abcam, anti-cytokeratin was from Invitrogen and anti-actin was from Biosource. Mouse antibodies against MHCI were from Biolegend, anti-Lamp1 from Abcam, Rabbit antibodies anti-EEA1 were from Novus Biologicals. Rabbit anti-VAP-A antibody was kindly provided by Antonella De Matteis, (Telethon Institute of Genetics and Medicine, Pozzuoli, Naples) and rabbit anti-Giantin was from Covance. Alexa 488- and Alexa 568-conjugated secondary anti-rabbit or anti-mouse goat antibodies were from Molecular Probes. Recombinant NarE was conjugated with fixable Alexa-Fluor-546 according to manufacturers’ instructions (kit A10237, Life Technologies). DNase I, Fluorescent Deoxyribonuclease Conjugates, Phalloidin-TRIC as well as FITC-labeled dUTP (Click-iT TUNEL Alexa Fluor 488 Imaging Assay) were obtained from Molecular Probes. CHX and NAD were purchased from Calbiochem (Merck Biosciences GmbH, Schwalboch, Germany). NarE (accession number NMB1343) and NarE-R7K recombinant proteins were cloned, expressed and purified as described [3]. In both preparations the endotoxin content, determined using a LAL assay (QCL-1000 kit from Lonza), was below 0.5 EU/mg protein.

### Ethics statement

Mouse studies were performed in accordance with Novartis Animal Welfare Policies and Italian laws on care and protection of laboratory animals. Mice were euthanized in accordance with experimental protocols. Experimental protocols were reviewed and approved by the Italian National Institute of Health and by the local Novartis Vaccines and Diagnostics Animal Welfare Body (authorization AEC 201101). CD1 mice (Charles River Laboratories) were used for the experiments.

### FACS analysis

To measure NarE binding to Chang cells, 2x10^5^ cells were incubated with different concentrations of NarE or NarE-R7K (ranging from 0,01 to 100 μg/ml) in serum-free DMEM in 96-well culture plates for 1h on ice. Cells were then washed and incubated on ice with anti-NarE serum for 30 min. After washing, cells were stained with FITC conjugated secondary antibodies. Cells were analysed with a Canto II flow cytometer (Beckton-Dickinson) by using Flowjo software. The geometric mean fluorescence intensity (MFI) for each population was calculated.

### In vitro ADP-ribosylation assay

For cell-free ADP-ribosylation assay, 50 μg of whole-cell lysate were used. ADP-ribosylation reaction was performed for 30 min at 37°C with 1 μg NarE or NarE-R7K in a buffer containing 20 mM Tris-HCl (pH 8), 1 mM EDTA, 1 mM DTT, 5 mM MgCl_2_, 10 μM biotinylated NAD^+^ (Trevigen) and Complete protease inhibitor (Roche, Basel, Switzerland) (according to the manufacturer’s manual). The reaction was stopped by adding 4x NuPAGE LDS Sample Buffer (Invitrogen) and 10x NuPAGE Reducing Agent (Invitrogen) and boiling the samples for 5 min at 100°C. The samples were subjected to SDS-PAGE and subsequently the biotin-ADP-ribosylated proteins were transferred to a nitrocellulose membrane and visualized with HRP-conjugated streptavidin.

### Immunofluorescence microscopy and pulse-chase experiments

Chang cells (5x10^4^/well) were plated on 8-wells Lab-Tek II Chamber Slide System (Thermo Scientific) and incubated overnight at 37°C 5% CO2. For NarE binding and to visualize morphological effects, cells were incubated at for different times with NarE/NarE-R7K. After washing with PBS, cells were fixed in paraformaldehyde 2% for 20 min at RT (room temperature) and then, after three washes with PBS, permeabilized with TritonX 0.1% plus saponin 1% for 20 min at RT. Cells were washed three times with PBST (PBS 1x, TritonX 0,1%) and incubated for 30 min at RT with Duolink II Blocking Solution 1X (Olink Bioscience). After washing with PBST twice, cells were incubated for 15 min at RT with mouse anti-NarE serum. Cells were washed again with PBST twice and incubated for 10 min at RT with secondary antibody Alexa Fluor 568 anti-mouse. Subsequently, cells were washed with PBS twice and, after drying at RT, one drop of ProLong Gold Antifade Reagent with DAPI (Invitrogen) was added. For pulse-chase experiments, in order to detach not internalized NarE bound to the plasma membrane of Chang cells, they were first incubated with NarE for 1 h, then they were washed with ice-cold phosphate-buffered saline (PBS) containing 500 mM NaCl and 0.5% acetic acid and three times with complete medium; before fixation the cells were incubated for 1 h. For the analysis of the subcellular localization of NarE, the slides were analyzed by Confocal microscopy, performed on a LSM700 (Carl Zeiss, Inc.) and LSM 510 (Carl Zeiss, Inc.) confocal microscopes using a Plan-Neofluar 63X objective. Detectors were set to detect an optimal signal below the saturation limits. Images were processed with Zen 2009 image software (Carl Zeiss, Inc.).

### xCELLigence system: time- and dose-dependent cell response profiles of Chang cells

The effect of NarE on epithelial barrier function was measured with an electrical cell-substrate impedance sensing system (Applied BioPhysics). In total, 1 × 10^5^ Chang cells were seeded in 8W10E cultureware and incubated at 37°C in a carbon dioxide incubator. Resistance of the monolayer was recorded until a stable resistance of approximately 600–1000 Ω was documented prior to the addition of purified protein.

### Cell treatments, lysis, and immunoblots

For immunoblot analysis, cells (2x10^6^ cells/well) were plated in a 6-well dish, grown overnight at 37°C to reach a sub-confluent condition, and incubated with NarE/NarE-R7K at 37°C in a humidified atmosphere with 5% CO2 for different time. Cells were recovered by centrifugation at 16,000Xg for 30 sec at 4°C, washed 2X in PBS and lysed in 1% (v/v) Triton X-100 in 20 mM Tris-HCl pH 8, 150 mM NaCl (in the presence of0.2 mg/ml Na orthovanadate, 1 mg/ml pepstatin, leupeptin, and aprotinin, and 10 mM phenyl methyl sulfonyl fluoride). Alternatively, for subcellular protein fractionation, cells were collected, washed in ice-cold PBS and processed by Thermo scientific subcellular protein fractionation Kit. Protein content of isolated fractions, corresponding to cytoplasmic, membrane, nuclear and pellet fraction, was determined by BCA assay. Equal amounts of proteins from were resolved by NuPage Novex 4–12% Bis-Tris Gels (Invitrogen) and transferred to 0.45-mm nitrocellulose filters nitrocellulose membrane by means of iBlot Gel Transfer Device (Invitrogen). Pre-stained molecular mass markers (Invitrogen) were included in each gel. Immunoblots were carried out using primary antibodies and peroxidase-labeled secondary antibodies according to the manufacturers’ instructions and a chemiluminescence detection kit (Pierce). Blots were scanned using a laser densitometer (DuoscanT2500 Agfa, Milan, Italy) and quantified using the ImageQuant5.0 software (Molecular Dynamics, Sunnyvale, CA). Data were normalized to loading controls.

### Statistical analysis

Mean values, standard deviation values and Student’s t test (unpaired) were calculated using the Microsoft Excel application. A level of P <0.05 was considered statistically significant.

## Results

### NarE binding to human epithelial cells leads to protein internalization

**Fig 1:**
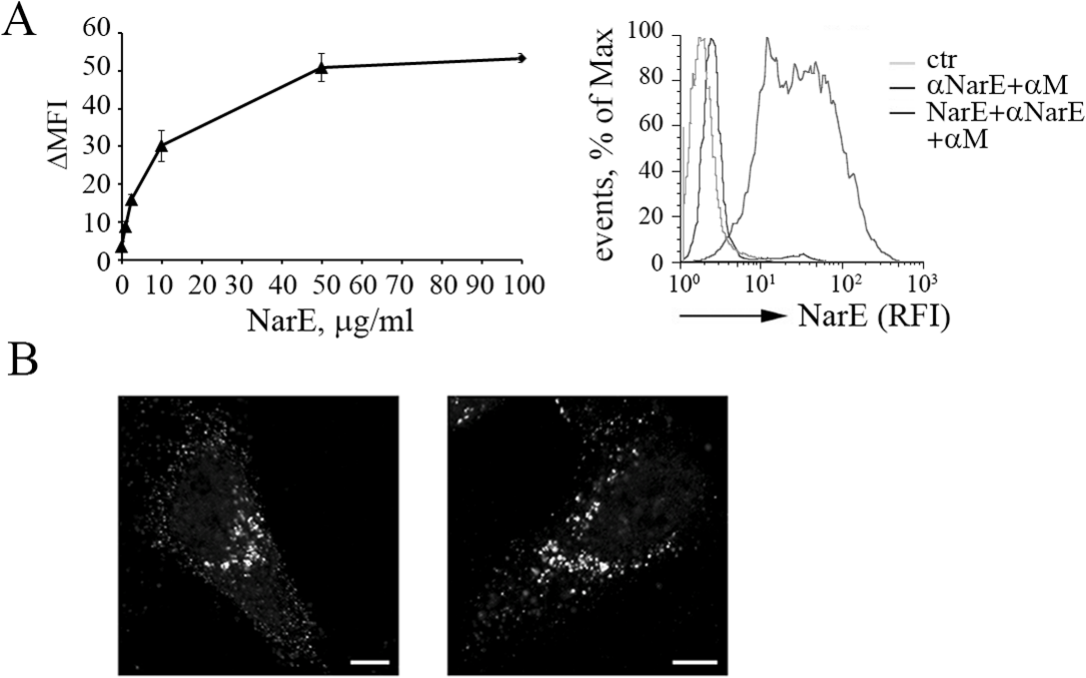
NarE binding to human epithelial cells leads to protein internalization. (A) Flow cytometric analysis of NarE binding to Chang cells for 1h at 4°C. The data are expressed as the difference between the MFI of cells incubated with NarE, anti-NarE antibodies (α-NarE), anti–mouse Ig FITC-labelled secondary antibodies (α-M), and the MFI of cells incubated with primary and secondary antibodies in the absence of NarE (ΔMFI). A representative concentration response curve (*n* > 3) and FACS profile (NarE 1μg/ml) are shown. RFI, relative fluorescence intensity. (B) Chang cells were plated on coverslips and incubated with 1 g/ml of recombinant NarE for 1 h. Next, the cells were washed with a high-salt and acid buffer to rinse the non-internalized NarE. Finally, the cells were fixed and stained with an anti-NarE antibodies (Fig 1B, *left panel*) or further incubated for 1h at 37°C before being processed (Fig 1B, *right panel*). All the images were acquired using with the same imaging conditions. Three independent experiments were performed. Scale bar is 10 μm.

The biological function of NarE was assessed on Chang human epithelial cells, a well-established *in vitro* model to study *N*. *meningitidis* interaction with the host [21, 22]. Purified recombinant protein was added at increasing concentrations to epithelial cells and the binding was measured by flow cytometric analysis. As shown in Fig 1A, NarE, is able to bind Chang cells in a concentration dependent manner, with a rapid increase in binding at lower concentrations, a kinetic probably reflecting NarE interaction with a high affinity receptor. Similar results were obtained when the enzymatically inactive variant of the toxin, NarE-R7K, which is devoid of the ADP-ribosylation activity, was used [23] (data not shown). The evidence that NarE is able to bind to host epithelial cells was further demonstrated by confocal analysis (S1 Fig). Of interest, we observed that NarE not only is able to bind to target cells but it can also be internalized. Indeed, confocal microscopy analysis of cells extensively washed with high-salt acidic buffer (which allows the removal of surface-associated proteins) revealed that NarE is found intracellularly within numerous perinuclear structures (Fig 1B).

### NarE is internalized through an endocytic traffic route

**Fig 2:**
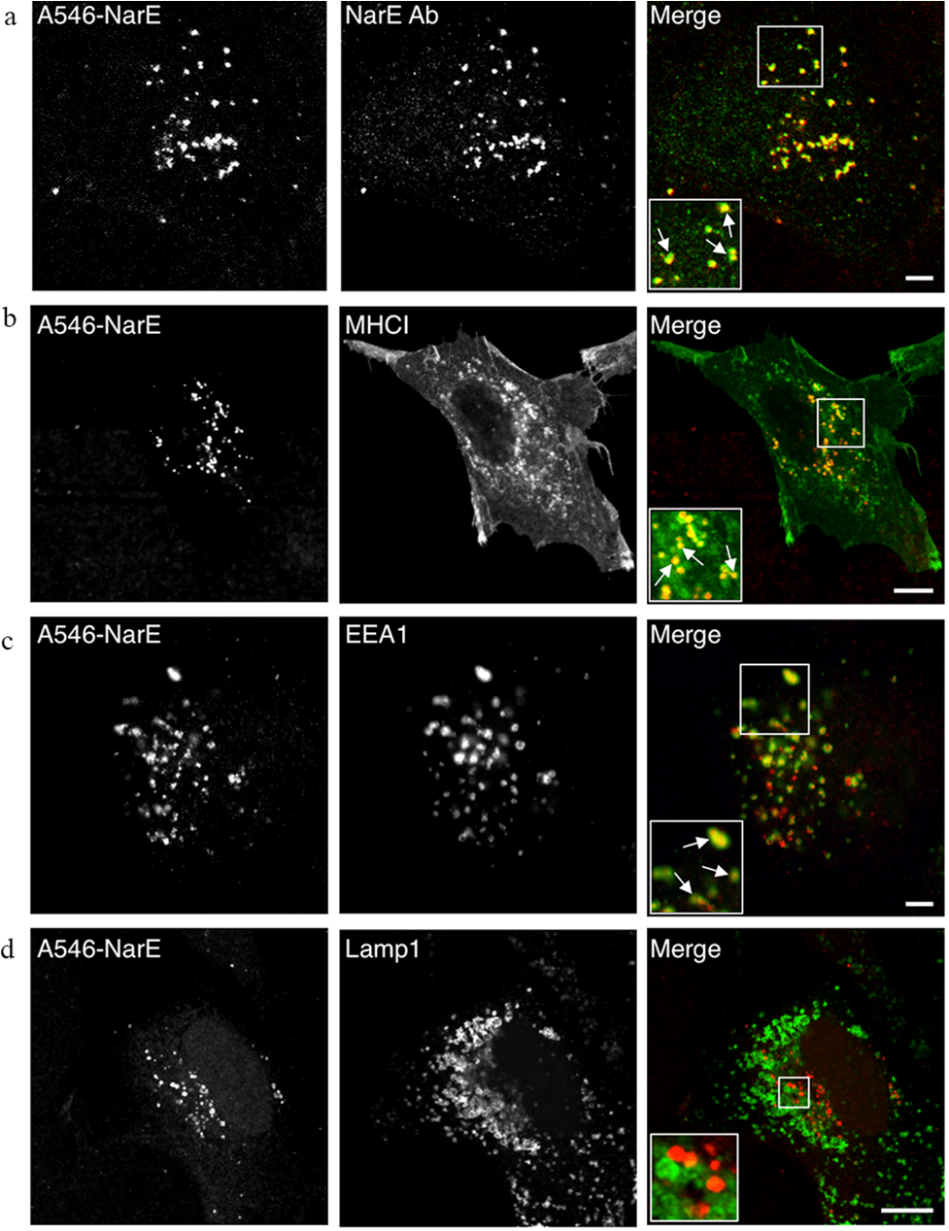
Immunofluorescence analysis of NarE subcellular localization. Chang cells were plated on coverslips and incubated for 1 h with (1 μg/ml) Alexa 546-NarE. Then, the cells were fixed and labelled with antibodies against NarE (a), MHCI (b), EEA1 (c) and Lamp1 (d). Areas of co-localization of Alexa 546-NarE with the signals detected using antibodies against NarE, MHCI and EEA1 are magnified in the inserts and highlighted by white arrows. Scale bar is 10 μm.

To further characterize the intracellular trafficking of NarE, Chang cells were incubated with recombinant fluorescently labelled NarE (Alexa 546-NarE) for 1h at 37°C. Cells were then processed by immunofluorescence analysis to assess the co-localization of NarE with established markers of intracellular compartments. Confocal analysis showed that NarE is distributed in punctate structures at the peripheral and juxtanuclear region of the cell (Fig 2A). Notably, these structures were found to co-localize with the major histocompatibility complex Class I (MHCI) protein (Fig 2B), a marker of recycling endosomes, and EEA1 (Fig 2C), a marker of early endosomes. No co-localization was observed with Golgi and ER specific markers, or with LAMP-1, a lysosomal-associated membrane protein (S2 Fig and Fig 2D, respectively). These data postulate that NarE is actively internalized into epithelial cells through an endocytic traffic route.

### NarE gains access to the cytoplasm and targets host cell proteins

**Fig 3:**
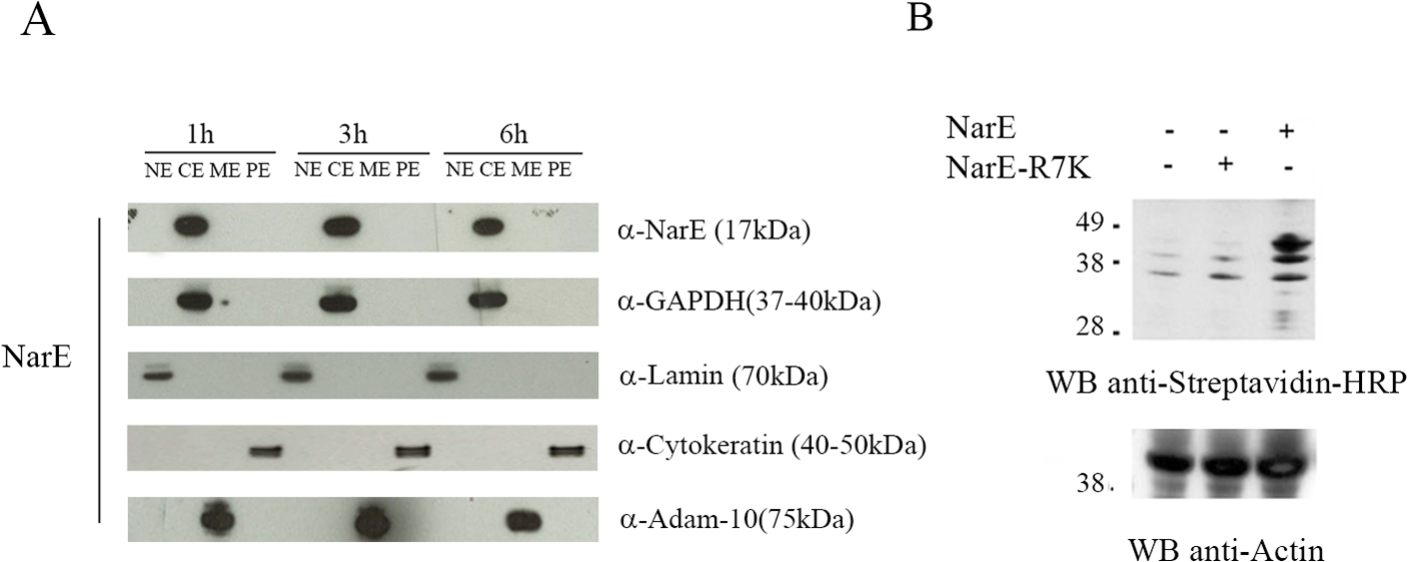
NarE gains access to the cytoplasm and targets host cell proteins. (A) Chang cells, grown to confluence, were exposed to NarE (1μg/ml) or saline (control) for 1 h washed and further incubated for 1, 3 and 6h. At the end of the incubation period cytoplasmic (CE), membrane (ME), nuclear soluble (NE) and cytoskeletal (PE) fractions were isolated and processed for western blot analysis. A mouse polyclonal anti-NarE antibody was used. Polyclonal antibodies anti-GAPDH, anti-ADAM10, anti-Lamin and anti-Cytokeratin were used to check the purity of each fraction. HRP-conjugated secondary antibodies were used before developing in chemiluminescence. The molecular weights of the single proteins are in brackets. (B) Immunoblot analysis of the ADP-ribosylation state of NarE substrates in whole-cell extracts from Chang cells treated with NarE (1μg/ml) for 30 min at 37°C. Biotin-ADP-ribose labelled proteins were identified using an anti-streptavidin HRP antibody. The results of a representative experiment are shown. Anti-actin was use as loading control. The position of the molecular mass markers are indicated on the left.

We then hypothesized that NarE internalization and consequent association to the endocytic route, may allow this ADP-ribosyl transferase to gain access to the cytosol. To this end we isolated cytoplasmic, membrane, nuclear soluble and cytoskeletal fractions from Chang cells incubated with NarE. Briefly, Chang cells were exposed to NarE for 1 h, washed, to remove unbound NarE, and further incubated for 1, 3 and 6h before being processed. As shown in Fig 3A, Western blotting analysis revealed that already at 2h, NarE accumulated in the cytosolic fraction. To identify putative cellular substrate/s ADP-ribosylated by NarE, whole-cell lysates were incubated with NarE or NarE-R7K in presence of biotinylated NAD^+^ (or with ^32^P-NAD^+^, data not shown). Interestingly, as shown in Fig 3B, incubation of cell extracts with NarE, but not with the genetically inactivated mutant NarE-R7K, resulted in the increase of ADP-ribosylated proteins, indicating that NarE targets Chang cell proteins. Of note, we detected bands of approximately 42–45 kDa, compatible with the molecular weight of actin, or of α-subunit of heterotrimeric G proteins, both well-known targets for modification of many bacterial toxins [8, 7].

### NarE impairs human epithelial monolayer integrity and induces cell rounding

**Fig 4:**
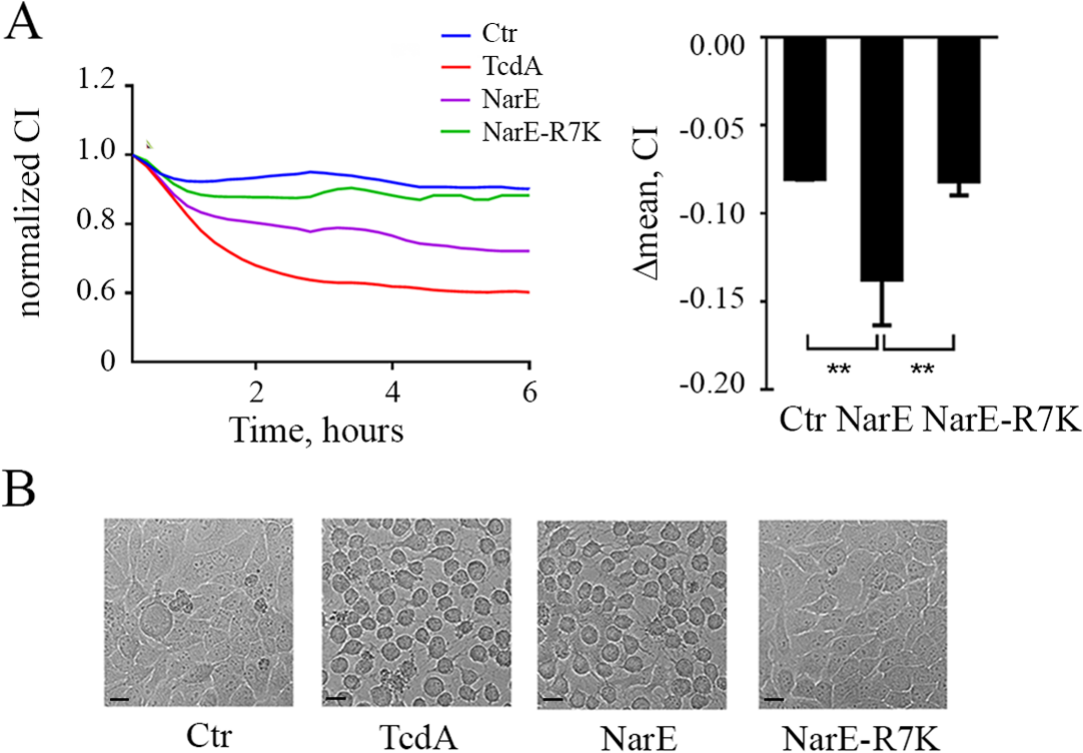
NarE impairs human epithelial monolayer integrity and induces cell rounding. (A) *Top left*, Chang monolayer was treated either with NarE or NarE-R7K (1μg/ml) and barrier resistance was continuously measured using an electric cell-substrate impedance sensing (xCELLigence) system. TcdA (100 ng/ml) was used as positive control, while saline was used as negative control. CI, Cell Index (arbitrary unit for electric impedance measurement). *Top right*, Variation of Chang cells CI after 6 h of treatment with NarE or NarE-R7K (1μg/ml). The results are expressed as the difference between normalized CI before agents addition (CI = 1) and CI value after 6 h of incubation averaged from three independent experiments, each performed on duplicate samples. Error bars represent the SD. **, P ≤ 0.01. (B) Effect of NarE treatment on the morphology of Chang cells. Chang cells were incubated at 37°C with NarE, NarE-R7K (1μg/ml), TcdA as positive control (100 ng/ml) or left untreated as control. After 4 hours, pictures were taken to assess the change in morphology. Bar, 20 μm.

Since NarE ADP-ribosylates Chang substrates, we then examined whether it could alter essential functions of eukaryotic cells. To this end electrical cell-substrate impedance sensing (xCELLigence) was used to measure trans-epithelial electrical resistance. Impedance reflects the status of the cell monolayer, including confluence, viability, and junction functionality [24, 25]. Chang cells were grown to confluence on xCELLigence inserts and the experiments were started when trans-epithelial resistance (TEER) was found constant between two measures taken 24 hours apart. Cells were then incubated with NarE or NarE-R7K. Vehicle was used as negative control. Changes in monolayer integrity were monitored every 15 min. As shown in Fig 4A Chang monolayer treated with 1μg/ml NarE demonstrated a rapid and progressive loss of resistance. Similar results were obtained when *Clostridium difficile* toxin, TcdA, a well-known cytotoxin affecting actin cytoskeleton [7, 26], was used. Neither vehicle nor NarER-7K altered monolayer resistance.

In the attempt to correlate changes in monolayer integrity with cytopathic events, NarE-R7K and NarE treated cells were visualized by light microscopy. As shown in Fig 4B NarE, as well as TcdA, our positive control, induced morphological changes as the cells rounded up within 4h (Fig 4B). On the contrary, host cells exposed to the mutant form of the protein did not show any changes in their morphology, indicating that induction of the cell-rounding phenotype of Chang cells required a functional ADP-ribosylating domain.

### NarE impacts on actin cytoskeleton

**Fig 5:**
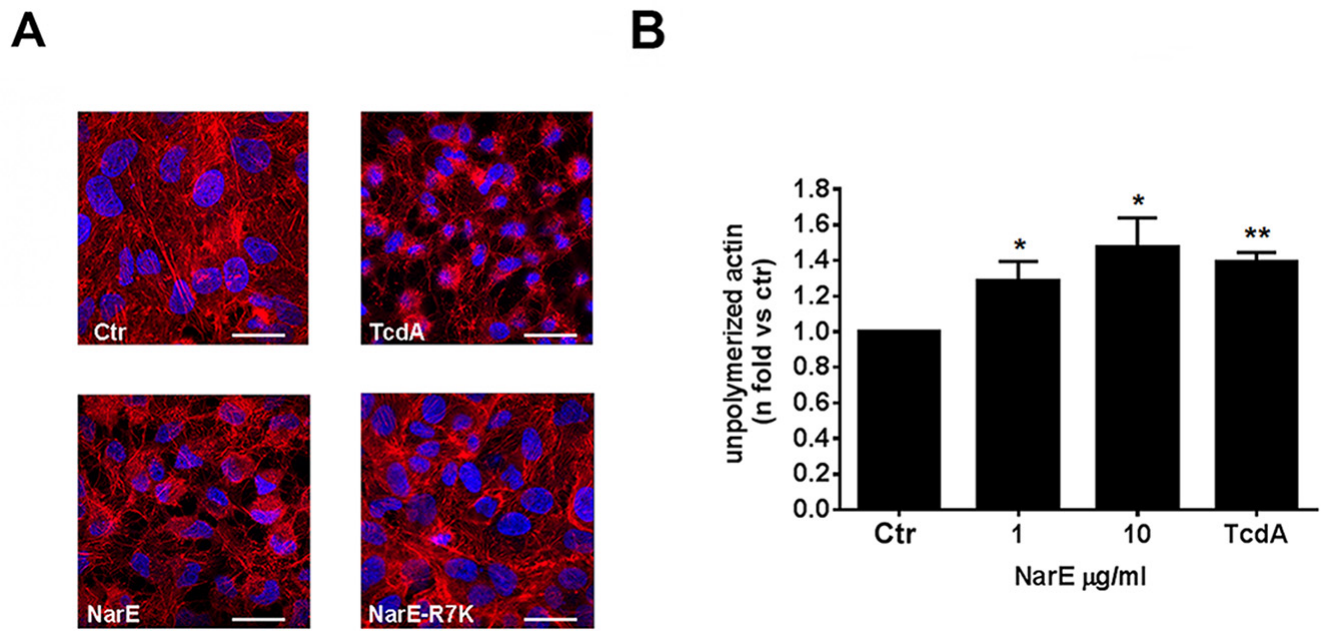
NarE impacts on actin cytoskeleton. (A) Actin-staining using TRITC-conjugated phalloidin (red) in Chang cells. The nucleus was stained with DAPI (blue). 1 μg/ml NarE, as well as 100 ng/ml TcdA, used as positive control, causes disruption of the actin cytoskeleton. Scale bar represents 20 μm. (B) Flow cytometric analysis of relative unpolymerized actin (fold increase vs untreated control, gray bar) in Chang cells treated with 1–10 μg/ml NarE or 100 ng/ml TcdA, as positive control. Data in columns are given from 3 independent experiments. **P≤0.01;*P≤0.05. Error bars, SD.

The observation that one of the ADP-ribosylated substrates could be actin (Fig 3, panel B), along with the phenotype of intoxication (cell rounding), lead us to further assess NarE effect on actin cytoskeleton of Chang cells. To this end, after 4 hours of incubation of cells with the wild-type or the mutant protein, morphological alterations were analyzed by confocal microscopy using fluorochrome-conjugated phalloidin. F-actin depolymerization and dissolution of cytoskeleton was observed in cells treated with NarE, suggesting that actin could be, directly or indirectly, a target of NarE-ADP-ribosylating activity. Representative micrographs are shown in Fig 5A. These results were further corroborated by monitoring global changes in actin dynamics using the DNase I inhibition assay, a selective analysis in which G-actin is detected with the fluorophore-derivatized Dnase I. Fig 5B displays a significant increase in the amount of unpolymerized actin after incubation with NarE. Similar results were obtained when TcdA was used. Notably, any effect on actin cytoskeleton was detected in cells treated with NarE-R7K.

### NarE induces apoptosis in Chang cells

**Fig 6:**
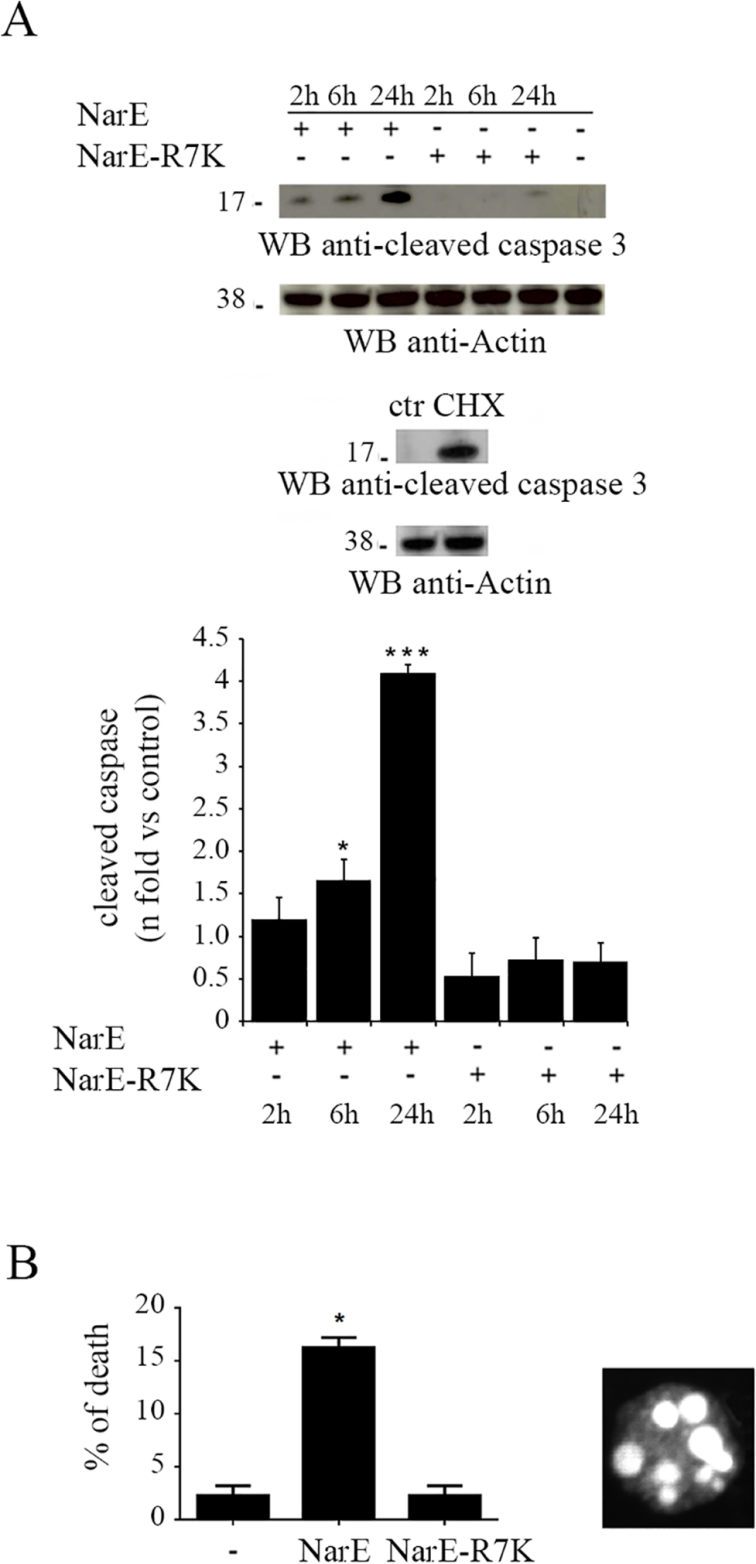
NarE induces apoptosis in Chang cells. (A) *Top panel*, Immunoblot analysis, using a specific antibody to detect caspases-3 cleavage, in post-nuclear supernatants from Chang cells incubated for the indicated times in the presence or absence of either 1 μg/ml NarE or NarE-R7K. *Bottom panel*, Quantification by laser densitometry of the relative levels of caspase-3 cleavage (fold activation *vs* untreated controls) in Chang cells in the presence of 1 μg/ml NarE or NarE-R7K (n = 3). CHX, cycloheximide, 10 μg/ml for 24h, (as positive control); ctr, control. *P≤0.05, ***P≤0.001. Error bars, SD. (B) Fluorescence microscopy analysis of apoptotic cells treated with NarE for 24 hours. The data are expressed as percentages of TUNEL-positive cells (*n* = 2; *P≤0.05; percentages were calculated on 100 cells/sample).

We next investigated whether NarE-induced cell damages correlate with the activation of cell death pathways. Among a wide range of factors controlling apoptotic cell death, caspase-3 activation plays a key role and its activation requires proteolytic processing of its inactive zymogene into activated p17/19 and p12 fragments [27]. Therefore in order to assess NarE effect on the apoptotic pathway, Chang cells were incubated with NarE, NarE-R7K or cycloheximide, CHX, (as positive control) for various times (2h to 24h) and then caspase-3 cleavage, which is an indication of its activation state, was evaluated. As reported in Fig 6, 2h and 6 h of incubation with the toxin were sufficient to induce caspase 3 cleavage. However, there was a peak activity after 24 h of treatment. As expected, NarE-R7K, which is devoid of the ADP-ribosylation activity, did not induce caspase-3 activation.

The development of apoptosis was further assessed using Terminal deoxynucleotidyltransferase-mediated dUTP-biotin nick end labeling (TUNEL). To this end Chang cells were treated with NarE for up to 24h. The appearance of fluorescence in the nuclei of NarE-treated cells showed evidence of apoptosis (Fig 6B, *right panel*), whereas no DNA fragmentation was observed in cells treated with NarE-R7K or in control cells (vehicle only). Following incubation with NarE for 24h, the amount of apoptotic cells significantly increased as shown by elevation of fragmented DNA (calculated as specific apoptosis as percentage of total cells) in Fig 6B (*left panel*).

## Discussion

In this paper, we focus on the biological functions of NarE when getting in contact to epithelial cells. In particular, we reported that NarE binds to Chang cells in a dose dependent and saturable manner, and that these events trigger the internalization of this ADP-ribosyltransferase that once in the cytoplasm, exerts its enzymatic activity on host cell proteins. This results in the alteration of cell morphology and epithelial monolayer integrity leading to the activation of caspase-3, an apoptosis executer (cytotoxic effect). Notably, similar cytopathic and cytotoxic effects are reported for various bacterial toxins targeting mammalian cells (e.g. *C*. *botulinum* C2, *C*. *perfringens* iota toxin and *S*. *enterica* SpvB), which by inducing loss of adhesion and cell rounding lead to the activation of the apoptotic pathway [28, 29, 7].

To demonstrate that ADP-ribosylating activity of NarE is crucial in inducing a toxic phenotype, a catalytically inactivated mutant, NarE-R7K, was used. As reported by Koehler C. et al. [23], direct mutagenesis of R7 residue, to K, results in a drastic reduction of ADP-ribosylation activity. The NarE mutant loses both transferase and glycohydrolase activities, likely due to a reduced NAD binding.

ADP-ribosyltransferase activities have been observed in many prokaryotic and eukaryotic species and in viruses. The best characterized mono-ADP-ribosylation reactions are those catalyzed by bacterial toxins, such as diphtheria, cholera, pertussis and clostridial toxins, which act by modifying crucial host cell proteins such as the α-subunit of heterotrimeric GTP-binding (G) proteins, the small GTPase Rho, monomeric actin and elongation factor 2 (EF-2), resulting in permanent activation or inactivation of the cell functions modulated by these protein substrates. Consequently, these toxins have been characterized extensively for their activity and represent some of the best understood microbial mediators of disease and have been used as components of vaccines able to prevent associated diseases [30, 31].

By *in silico* studies Masignani and colleagues identified an ADP-ribosyltransferase in MC58 strain of *N*. *meningitidis* serogroup B. They demonstrated that this protein is able to hydrolase NAD and to transfer the ADP-ribose group to small guanidine compounds like agmatine and arginine and so they called it NarE, *N. meningitidis* ADP-ribosylating enzyme. Interestingly, they found that, despite the absence of a predicted leader peptide, NarE efficiently accumulates in the periplasm of Meningococcus [3]. Further, they showed that the *narE* gene is present only in a subset of hypervirulent strains of bacteria.

An open question is how NarE, being a periplasmic protein, could achieve contact with host cells. NarE lacks a leader peptide as well as a gene coding for the translocation/receptor-binding subunit B but since it is a really small proteins it maybe can reach the periplasmic space of Meningococcus by translocating across the cytoplasmic membrane through natural membrane pores [3]. However, in order to exert a toxic activity, NarE has to contact host cells. For this reason, once in the periplasm, a second step is required to allow the release of the protein. A possible hypothesis would be that NarE follows a pathway similar to that described for LT of *E*. *coli* [32] and it could be released in association with vesicles. Internalization of vesicles could then allow intoxication of the host cells. Actually, we have experimental data suggesting that NarE is present in the outer membrane vesicles (OMVs) generated following treatment with denaturing agents of the MC58 strain (Rossi Paccani S. *et al*., unpublished observation). Furthermore, recent observations indicate that NarE protein is expressed in the OMVs of hypervirulent clones and that fusion of OMVs with host immune cell allows delivery of NarE, which impairs cellular effector function [33]. These data strongly support the hypothesis that NarE intoxication of host cells occurs upon internalization of OMVs. However, we cannot exclude that NarE export through the outer membrane could occur following cell contact or could just happen upon lysis of the bacterium [3].

Even if the mechanism by which NarE reaches host cells is still unknown, our data clearly highlight that the protein is able to bind to epithelial cells and alter their integrity suggesting a possible role of NarE in first stages of *N*. *meningitidis* pathogenesis, indeed disruption of epithelial barrier is a key step for a successful colonization of upper respiratory tract and spreading of infection. Further investigations are needed to elucidate the pathway followed by this newly identified ADP-ribosylating enzyme to get to epithelial cells and its potential role in the virulence and pathogenesis of meningococcal species.

## Supporting Information

S1 Fig NarE binds to Chang cells. Immunofluorescence analysis of NarE binding to Chang cells. Cells were either untreated or incubated with 1ug/ml NarE at 37°C (upper panel) or 4°C (lower panel) for 1 hour and then fixed and stained. Representative confocal images are shown. Cells were co-stained with anti-NarE (red), phalloidin (green) and DAPI (blue). Bar, 20 μm
(TIF)

S2 Fig Immunofluorescence analysis of NarE subcellular localization. Chang cells were plated on coverslips and incubated for the indicated times with NarE. Vehicle was used as a negative control. Cells were fixed and labelled with an anti-NarE antibody and with an anti-Giantin antibody (*left panel*) or an anti-VAP-A antibody (*right panel*). The red channel corresponds to NarE whereas the green one to Giantin or VAP-A. Magnified areas are shown in the inserts. All the images were acquired using the same confocal settings. Scale bar is 10 μm.
(TIF)
