# Supplementary figures and images for: The Neisseria meningitidis ADP-Ribosyltransferase NarE Enters Human Epithelial Cells and Disrupts Epithelial Monolayer Integrity
Source: PLoS One. 2015 May 21;10(5):e0127614. doi: 10.1371/journal.pone.0127614 (PMC4440719; doi:10.1371/journal.pone.0127614)

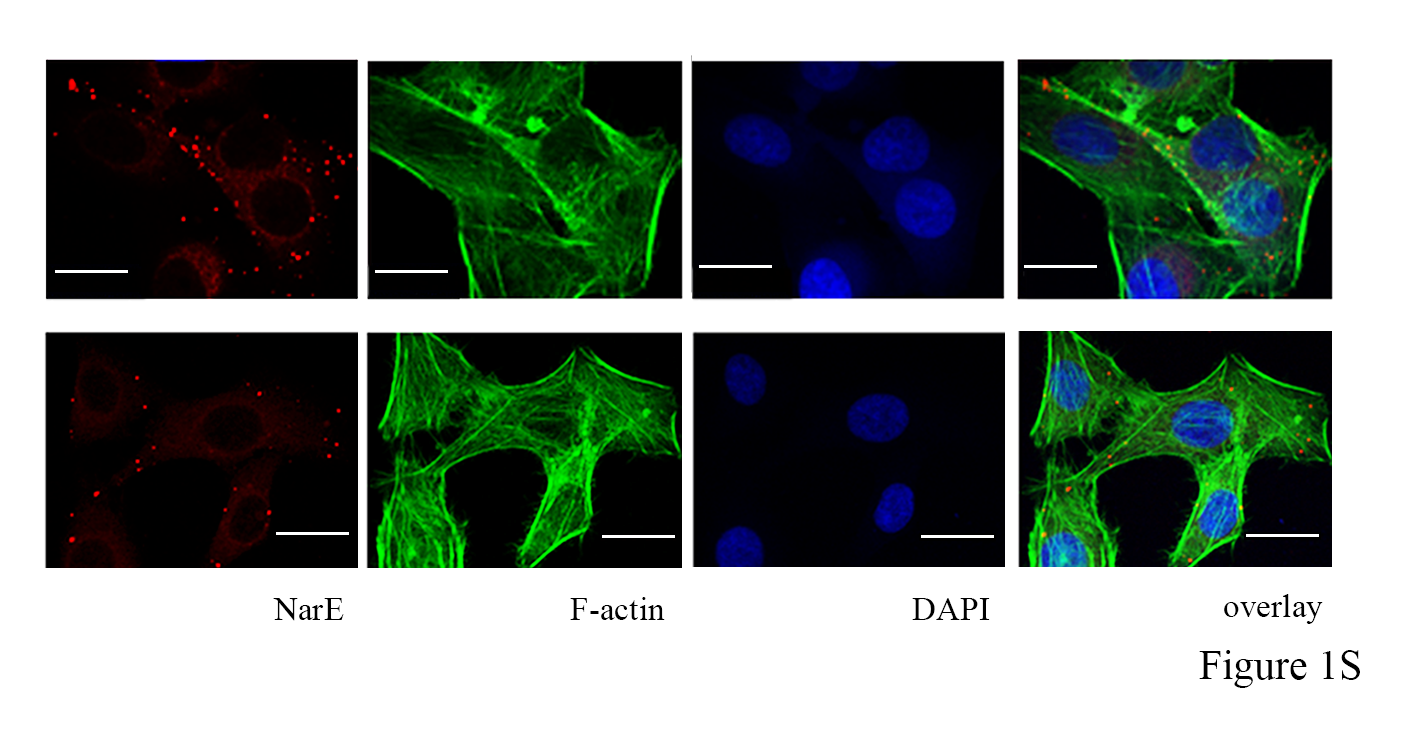

Supplement: S1 Fig — Immunofluorescence analysis of NarE binding to Chang cells. Cells were either untreated or incubated with 1ug/ml NarE at 37°C (upper panel) or 4°C (lower panel) for 1 hour and then fixed and stained. Representative confocal images are shown. Cells were co-stained with anti-NarE (red), phalloidin (green) and DAPI (blue). Bar, 20 μm (TIF) [file pone.0127614.s001.tif]

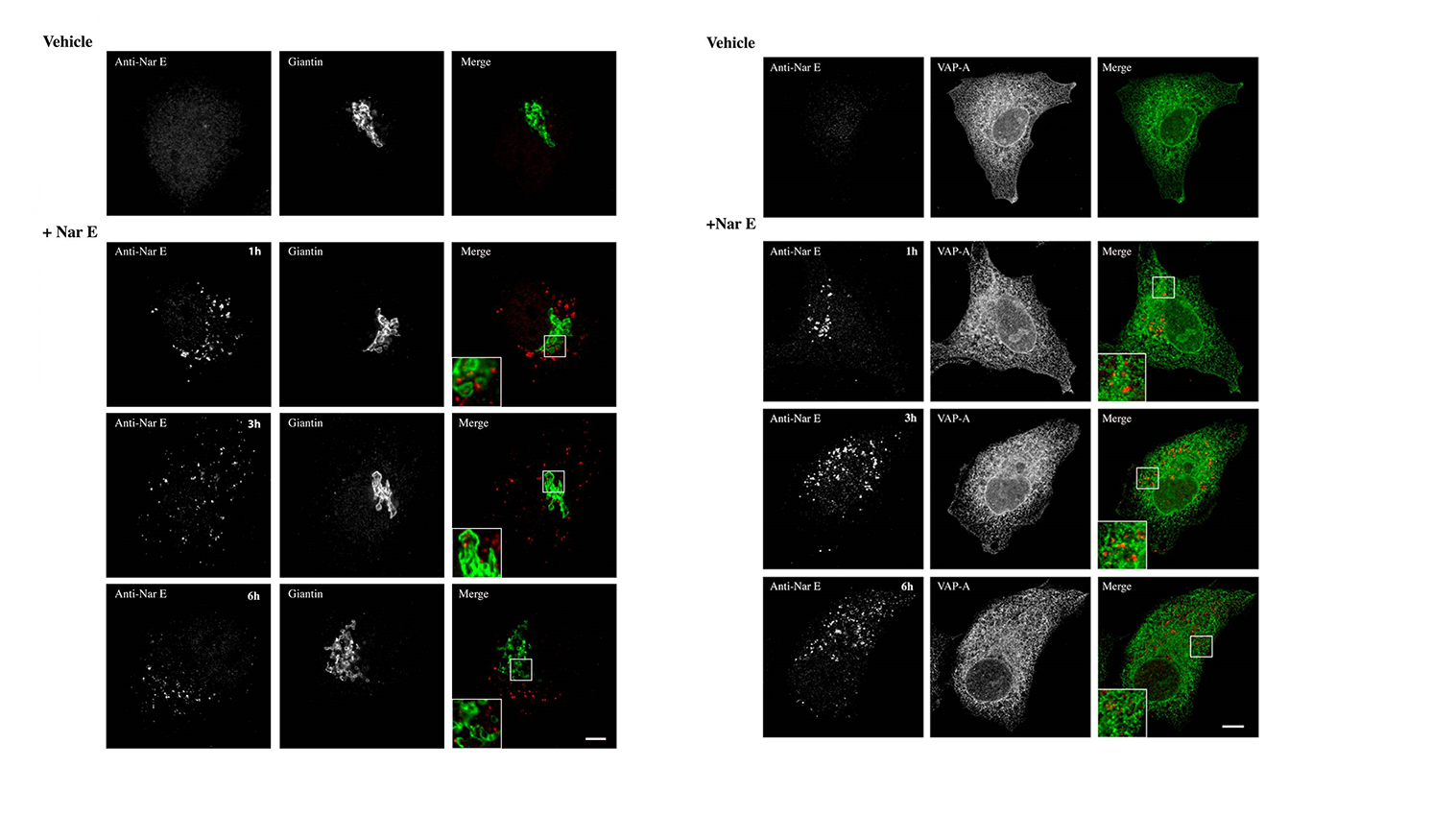

Supplement: S2 Fig — Chang cells were plated on coverslips and incubated for the indicated times with NarE. Vehicle was used as a negative control. Cells were fixed and labelled with an anti-NarE antibody and with an anti-Giantin antibody (left panel) or an anti-VAP-A antibody (right panel). The red channel corresponds to NarE whereas the green one to Giantin or VAP-A. Magnified areas are shown in the inserts. All the images were acquired using the same confocal settings. Scale bar is 10 μm. (TIF) [file pone.0127614.s002.tif]
